# Antioxidant Barrier and Oxidative Damage to Proteins, Lipids, and DNA/RNA in Adrenal Tumor Patients

**DOI:** 10.1155/2021/5543531

**Published:** 2021-06-22

**Authors:** Barbara Choromańska, Piotr Myśliwiec, Tomasz Kozłowski, Magdalena Łuba, Piotr Wojskowicz, Jacek Dadan, Hanna Myśliwiec, Katarzyna Choromańska, Anna Gibała, Anna Starzyńska, Małgorzata Żendzian-Piotrowska, Anna Zalewska, Mateusz Maciejczyk

**Affiliations:** ^1^Department of General and Endocrine Surgery, Medical University of Bialystok, 24a M. Sklodowskiej-Curie Street, 15-276 Bialystok, Poland; ^2^Department of Dermatology and Venereology, Medical University of Bialystok, 14 Żurawia Street, 15-540 Bialystok, Poland; ^3^Department of Oral Surgery, Medical University of Gdansk, 7 Dębinki Street, 80-211 Gdansk, Poland; ^4^Department of Hygiene, Epidemiology and Ergonomics, Medical University of Bialystok, 2c Mickiewicza Street, 15-233 Bialystok, Poland; ^5^Experimental Dentistry Laboratory, Medical University of Bialystok, 24a M. Sklodowskiej-Curie Street, 15-274 Bialystok, Poland

## Abstract

This study is the first to assess redox balance, glutathione metabolism, and oxidative damage to RNA/DNA, proteins, and lipids in the plasma/serum and urine of patients with adrenal masses. The study included 70 patients with adrenal tumors divided into three subgroups: incidentaloma (*n* = 30), pheochromocytoma (*n* = 20), and Cushing's/Conn's adenoma (*n* = 20), as well as 60 healthy controls. Blood and urine samples were collected before elective endoscopic adrenalectomy. Antioxidant defense capacity was significantly decreased (serum/plasma: superoxide dismutase (SOD), catalase (CAT) and reduced glutathione (GSH), uric acid (UA); urine: SOD, GSH, UA) in patients with adrenal masses. The oxidative damage to proteins (advanced glycation end products (AGE), advanced oxidation protein products (AOPP)) and lipids (lipid hydroperoxides (LOOH), and malondialdehyde (MDA)) was higher in the plasma and urine of these patients. Plasma MDA and DNA/RNA oxidation products, with high sensitivity and specificity, can help to diagnose pheochromocytoma. This biomarker differentiates patients with pheochromocytoma from Cushing's/Conn's adenoma as well as from heathy controls. Plasma RNA/DNA oxidation was also positively correlated with urine metanephrine. Oxidative stress can play a crucial role in adrenal tumors. However, further studies are required to clarify the role of redox signaling in adrenal masses.

## 1. Introduction

Adrenal masses are the most common of all tumors in humans [1, 2]. Most adrenal tumors are benign adenomas found incidentally in imaging studies of the abdominal cavity and commonly defined as adrenal incidentalomas [3, 4]. Although most adrenal gland lesions are benign adrenal cortex tumors and do not secrete hormones, a significant proportion may be hormonally active, and their clinical manifestation depends on the type of hormones secreted by the tumor. They can secret glucocorticosteroids (cortisol), leading to Cushing's syndrome and mineralocorticosteroids (aldosterone) and Conn's syndrome [2, 5]. Pheochromocytomas, accounting for approx. 5-7% of all adrenal tumors, originate from the adrenal medulla and secrete catecholamines [6]. Hormonal overproduction can be associated with severe morbidity and, in some cases, can lead to mortality. Moreover, it was found that the size of the tumor is related to the risk of malignancy. Tumors in the size range of 4.1 to 6 cm have a 6% malignancy risk, while tumors larger than 6 cm may be malignant in 25% of cases [7]. Malignant tumors include adrenocortical carcinoma (rare, though, very aggressive), malignant pheochromocytoma, and metastases. The clinical symptoms of adrenal tumors are not specific, and their diagnosis is often difficult. Therefore, a better to understanding of adrenal tumors' biology could help in search for new diagnostic biomarkers. Due to the variety of adrenal tumors, their pathogenesis is still not fully understood. Probably, genetic determinants play a vital role in the development of adrenal tumors. Known risk factors for adrenal tumor comprise smoking in men and oral hormonal contraceptives in women. The likelihood of adrenal tumors also increases among people with obesity, type 2 diabetes, and hypertension [8].

The role of oxidative stress (OS) in the development of obesity and its complications has already been established [9–12]. Recent studies emphasize the critical role of OS in the development of tumors [13–16]. In this process, overproduction of reactive oxygen species (ROS) and nitrogen (RNS) with a simultaneous reduction in enzymatic and nonenzymatic antioxidant protection was observed [17, 18]. Redox imbalance may lead to initiation, progression, and growth of tumor cells by activating redox-responsive signaling cascades [19]. It is well known that ROS induce peroxidation of lipids and proteins, leading to highly cytotoxic oxidation products for the cell [20]. Protein oxidation products accumulate in cells, inhibit proteasomes' activity, and cause further structural and functional damage to cell organelles [21]. Moreover, oxidative stress can damage DNA, contribute to cell death through necrosis, and inhibit apoptosis [22]. Although the antioxidant/oxidative homeostasis is disrupted in cancers, antioxidant systems protect cells from OS's damaging effects and repair some oxidative damage to biomolecules. Unfortunately, nothing is known about the effectiveness of the antioxidant barrier in patients with adrenal tumors. It is unclear whether disorders in redox mechanisms are associated with adrenal masses. Therefore, our study is aimed at assessing the enzymatic and nonenzymatic antioxidant barrier, glutathione metabolism, and oxidative damage to lipids, proteins, and DNA/RNA in the plasma, serum, and urine of patients with adrenal mass compared to the healthy controls. We are also the first to compare redox homeostasis between adrenal incidentalomas, pheochromocytomas, and Cushing/Conn adenoma.

## 2. Materials and Methods

The study was designed, conducted, and reported according to the guidelines for Good Clinical Practice and the Declaration of Helsinki and approved by the Bioethics Committee of the Medical University of Bialystok (permission numbers: R-I-002/66/2015, APK.002.341.2020). All patients participating in the study gave their informed consent.

The study included 70 patients (37 women and 33 men aged from 50 to 65 years) with adrenal masses diameter > 1 cm and<8 cm, who underwent elective endoscopic adrenalectomy using either lateral transperitoneal approach or posterior retroperitoneal approach. Patients were diagnosed at internal medicine departments with an endocrinology profile and treated surgically at the 1st Department of General and Endocrine Surgery at the University Hospital in Bialystok, Poland. The study group was divided into three subgroups: patients with incidentaloma (*n* = 30), pheochromocytoma (*n* = 20), and adenoma (*n* = 20). In the adenoma subgroup, 11 patients were diagnosed with Cushings', and 9 patients were diagnosed with Conns' syndrome. Patients with suspected phaeochromocytoma were treated with doxazosin—a selective alpha-1-adrenergic receptor blocker, for 10-14 days before surgery to avoid intraoperative hypertensive crisis. Patients with Conn's syndrome were supplemented with potassium or took spironolactone—an aldosterone receptor blocker in the preoperative period.

The control group consisted of 60 healthy individuals (31 women and 29 men aged from 50 to 65 years) who underwent a dental follow-up visit at the Specialist Dental Clinic at the Medical University of Bialystok. Only people with blood counts and biochemical blood test (Na +, K +, INR, AST, ALT and creatinine) values within the reference range were qualified to the control group.

The exclusion criteria for both study and control groups were acute inflammation, neoplastic diseases, metabolic diseases (insulin resistance, type 1 and type 2 diabetes, osteoporosis, gout, and mucopolysaccharidosis), autoimmune diseases (including ulcerative colitis, Hashimoto's disease, and Crohn's disease), cardiovascular diseases (other than hypertension in the study group), diseases of the respiratory, digestive and genitourinary systems, infectious diseases (HIV/AIDS, hepatitis A, B, and C), smoking, and alcohol abuse, as well as pregnancy in women.

Three months prior to collecting the material for the study, the patients from the study and control groups declared not taking nonsteroidal anti-inflammatory drugs, glucocorticosteroids, antibiotics, and vitamins and antioxidant supplements. The clinical characteristics of the study and control groups are demonstrated in Table 1.

### 2.1. Blood and Urine Collection

All samples were collected in a fasting state from patients with adrenal mass and healthy individuals who did not perform intense physical exercise twenty-four hours prior to blood sampling. Blood was collected into serum and EDTA tubes (S-Monovette SARSTEDT) and centrifuged at 4000 rpm for 10 minutes at 4°C. Urine for testing was collected in a sterile disposable container, immediately after bedtime, from the first morning portion of urine from the middle stream. The urine sample was centrifuged for 5 minutes at 1500 pm. The supernatant was protected against oxidation (10 *μ*l of 0.5 M BHT/1 ml of serum/plasma and urine) and stored at -80°C until the final analysis [23, 24]. The samples were stored at -80°C for no longer than six months.

### 2.2. Laboratory Measurements

Serum Na +, K +, full blood count, glucose, aldosterone, and serum cortisol before 10 a.m., as well as urine methanephrine and normethanephrine, were quantified by using an Abbott analyzer (Abbott Diagnostics, Wiesbaden, Germany).

### 2.3. Redox Assays

All reagents used for the redox assays were from Sigma-Aldrich (Nümbrecht, Germany/Saint Louis, MO, USA). Antioxidant enzymes were determined in serum. The nonenzymatic antioxidants, redox status, and oxidation products were evaluated in the plasma. The 96-well microplate reader BioTek Synergy H1 (Winooski, VT, USA) was used to measure absorbance/fluorescence. All determinations were conducted in duplicate samples and standardized to 1 mg of the total protein. The total protein content was assayed colorimetrically by the bicinchoninic acid assay with bovine serum albumin as a standard (Thermo Scientific PIERCE BCA Protein Assay Kit, Rockford, IL, USA).

### 2.4. Antioxidant Barrier

The activity of serum Cu-Zn-superoxide dismutase (SOD, EC 1.15.1.1) was evaluated spectrophotometrically at 480 nm by measuring the inhibition rate of adrenaline oxidation [25]. One unit of SOD activity was qualified as the amount of enzyme inhibiting adrenaline oxidation by 50%. The activity of serum catalase (CAT, EC 1.11.1.6) was determined by measuring at 240 nm spectrophotometrically hydrogen peroxide (H2O2) decomposition [26]. One unit of CAT activity was qualified as the quantity of the enzyme catalyzing decomposition of 1 mM of H_2_O_2_ per 1 min. The activity of serum glutathione peroxidase (GSH-Px, EC 1.11.1.9) was evaluated spectrophotometrically at 340 nm by measuring the reduction of organic peroxides by GSH-Px in the presence of reduced nicotinamide adenine dinucleotide phosphate (NADPH) [27]. The activity of serum glutathione reductase (GR, EC 1.8.1.7) was assessed spectrophotometrically based on the decrease in NADPH absorbance at 340 nm [28]. One unit of GR activity was defined as the quantity of enzyme catalyzing the oxidation of 1 *μ*M NADPH per 1 min.

The concentration of plasma uric acid (UA) was evaluated spectrophotometrically using the commercial kit (QuantiChromTM Uric Acid DIUA-250; BioAssay Systems, Harward, CA, USA), according to the manufacturer's instructions. The absorbance was measured at 630 nm. The concentration of plasma glutathione was determined colorimetrically at 412 nm based on the enzymatic reaction NADPH, 5,5′-dithiobis-(2-nitrobenzoic acid) (DTNB) and GR [29]. The reduced glutathione (GSH) concentration was counted from the difference between the concentration of total glutathione and oxidized glutathione (GSSG). Redox status was calculated according to the formula = [GSH]^2^/[GSSG] [30].

### 2.5. Oxidative Stress Products

The concentration of malondialdehyde (MDA) was assessed colorimetrically with the TBARS method using thiobarbituric acid (TBA). 1,1,3,3-Tetraethoxypropane was used as the standard, and determination was performed at a 535 nm wavelength [31]. The concentration of lipid hydroperoxides (LOOH) was measured spectrophotometrically with the FOX-2 test using the reaction of iron (3+) ions with xylenol orange (XO) [32, 33]. The absorbance of the Fe-XO complex was measured at a 560 nm wavelength. The content of plasma advanced glycation end products (AGE) was determined spectrofluorimetrically at 350/440 nm by measuring AGE-specific fluorescence [34]. The concentration of plasma advanced oxidation protein products (AOPP) was evaluated spectrophotometrically by measuring the plasma's iodide ion oxidizing capacity at 340 nm [34]. Immediately before the assay of AGE and AOPP, plasma was diluted (1 : 5, v:v) in 0.02 M PBS, pH 7.4 [35]. The concentration of plasma DNA/RNA oxidative damage was assayed according to the manufacturer's instructions, using commercial high sensitivity ELISA kits (DNA/RNA oxidative damage ELISA Kit, Cayman Chemicals, Ann Arbor, MI, USA, respectively). The test detects all three oxidized guanine species; 8-hydroxy-2′-deoxyguanosine from DNA, 8-hydroxyguanosine from RNA, and 8-hydroxyguanine from either DNA or RNA. The assay has a range from 10.3 to 3,000 pg/ml and a sensitivity of approximately 30 pg/ml.

### 2.6. Statistical Analysis

The statistical significance level was set at *p* < 0.05. The normality of the distribution was assessed using the Shapiro–Wilk test, while homogeneity of variance used the Levene test. For comparison of quantitative variables, the Kruskal–Wallis ANOVA test and Dunn's posthoc test were used. Multiplicity adjusted *p* value was also calculated. The relationship between the assessed redox biomarkers was evaluated using the Spearman rank correlation. Statistical analysis was performed using GraphPad Prism 8.3.0 for macOS (GraphPad Software, Inc. La Jolla, USA).

The number of subjects was determined based on our previous experiment, assuming that the test's power would be equal to 0.9 (online *ClinCalc* sample size calculator).

## 3. Results

Table 1 shows a comparison of the clinical and routine laboratory characteristics of the controls, incidentaloma, pheochromocytoma, and Cushing's/Conn's adenoma patients. We found significantly higher BMI and serum glucose values in patients with adrenal masses compared to the healthy controls. Urinary metanephrine and normetanephrine were greater in the pheochromocytoma group than any other group. In contrast, aldosterone was increased in Cushing's/Conn's adenoma group as compared to the controls. The PLT content was lower in patients with incidentaloma and Cushing's/Conn's adenoma than in the controls.

### 3.1. Superoxide Dismutase (SOD)

**Figure 1:**
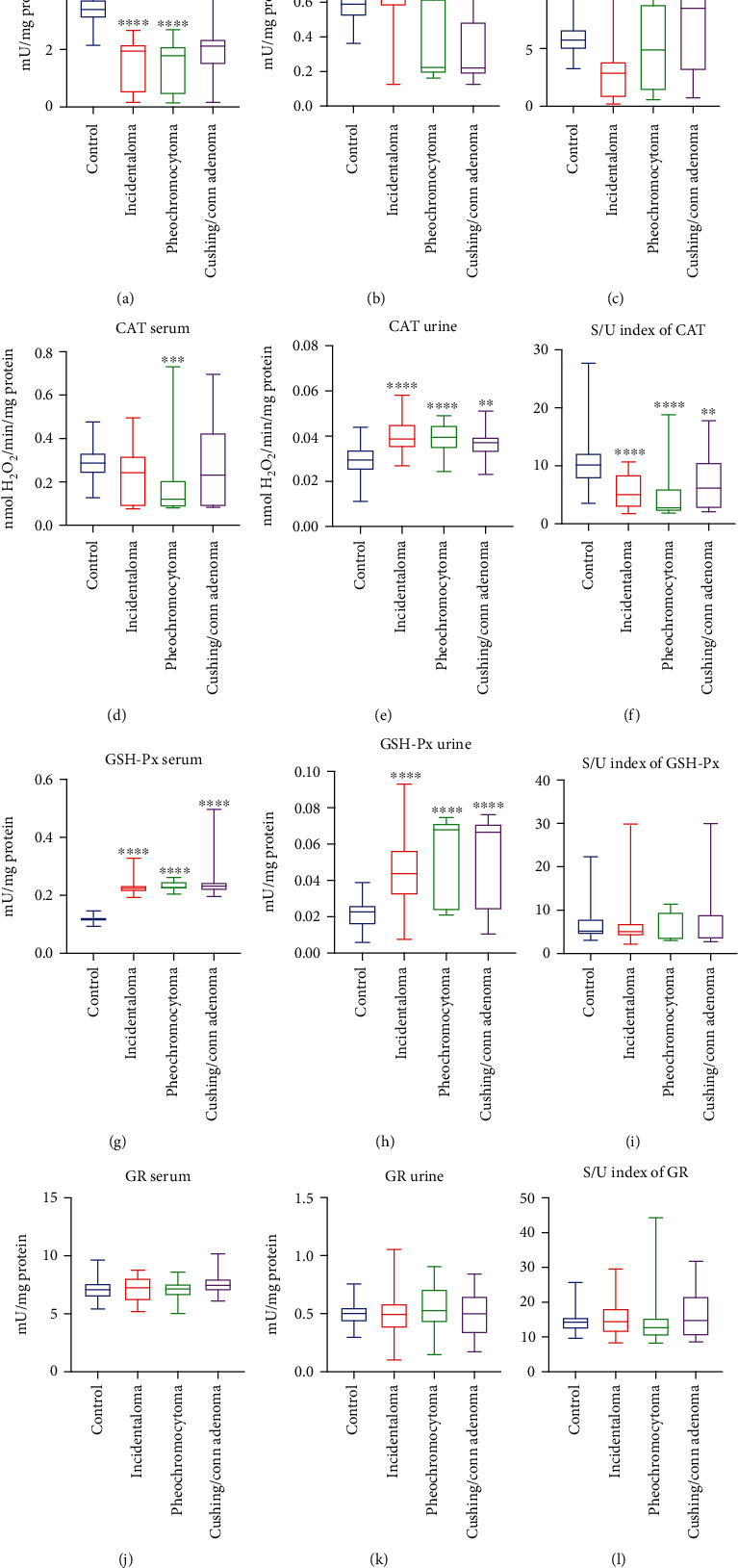
The activity of serum and urine enzymatic antioxidants (a, b, d, e, g, h, j, k), as well as serum/urine index of enzymatic antioxidants activity (c, f, i, l) of the controls, incidentaloma, pheochromocytoma, and Cushing's/Conn's adenoma patients. Results are presented as median with minimum and maximum. ^∗∗^*p* < 0.01, ^∗∗∗^*p* < 0.001, and ^∗∗∗∗^*p* < 0.0001 indicate significant differences from the controls; ^^*p* < 0.01 and ^^^*p* < 0.001 indicate significant differences from the pheochromocytoma group; SOD: superoxide dismutase; CAT: catalase; GSH-Px: glutathione peroxidase; GR: glutathione reductase (GR).

The activity of serum SOD was significantly decreased in all studied groups: incidentaloma (-43%, *p* < 0.0001), pheochromocytoma (-47%, *p* < 0.0001), and Cushing's/Conn's adenoma (-37%, *p* < 0.0001) as compared to the healthy controls (Figure 1(a)). urine SOD activity was markedly lower in pheochromocytoma (-62%, *p* = 0.0052) and Cushing's/Conn's adenoma (-63%, *p* < 0.0001) groups than the controls. Moreover, incidentaloma urine SOD activity was higher than pheochromocytoma (+193%, *p* = 0.0002) and Cushing's/Conn's adenoma (+197%, *p* < 0.0001) groups (Figure 1(b)). Additionally, the serum/urine index of SOD activity in incidentaloma (-50%, p <0.0001) was diminished in comparison with the controls. Interestingly, the serum/urine index of SOD activity in incidentaloma was lower than pheochromocytoma (-41%, *p* = 0.0064) and Cushing's/Conn's adenoma (-64%, *p* < 0.0001) groups (Figure 1(c)).

### 3.2. Catalase (CAT)

We found lower serum activity of CAT only in patients with pheochromocytoma (-58%, *p* < 0.001) in comparison with the controls (Figure 1(d)). The activity of urine CAT was significantly increased in all studied groups: incidentaloma (+31%, *p* < 0.0001), pheochromocytoma (+34%, *p* < 0.0001), and Cushing's/Conn's adenoma (+26%, *p* = 0.0016) as compared to the healthy controls (Figure 1(e)), whereas serum/urine index of CAT activity was decreased: incidentaloma (-50%, *p* < 0.0001), pheochromocytoma (-73%, *p* < 0.0001), and Cushing's/Conn's adenoma (-39%, *p* = 0.0092) (Figure 1(f)).

### 3.3. Glutathione Peroxidase (GSH-Px)

We observed greater serum and urine activity of GSH-Px in patients witch adrenal masses: incidentaloma (+90%, *p* < 0.0001; +92%, *p* < 0.0001), pheochromocytoma (91 + %, *p* < 0.0001; +198%, *p* < 0.0001), and Cushing's/Conn's adenoma (+96%, *p* < 0.0001; +192%, *p* < 0.0001) as compared to the controls (Figures 1(g) and 1(h)), while serum/urine index of GSH-Px activity did not differ between studied groups and the controls (Figure 1(i)).

### 3.4. Glutathione Reductase (GR)

There were no statistically significant differences in the activity of serum and urine GR and serum/urine index of GR activity in studied groups (incidentaloma, pheochromocytoma, and Cushing's/Conn's adenoma) compared with the controls (Figures 1(j), (k), and (l)).

### 3.5. Total Glutathione

**Figure 2:**
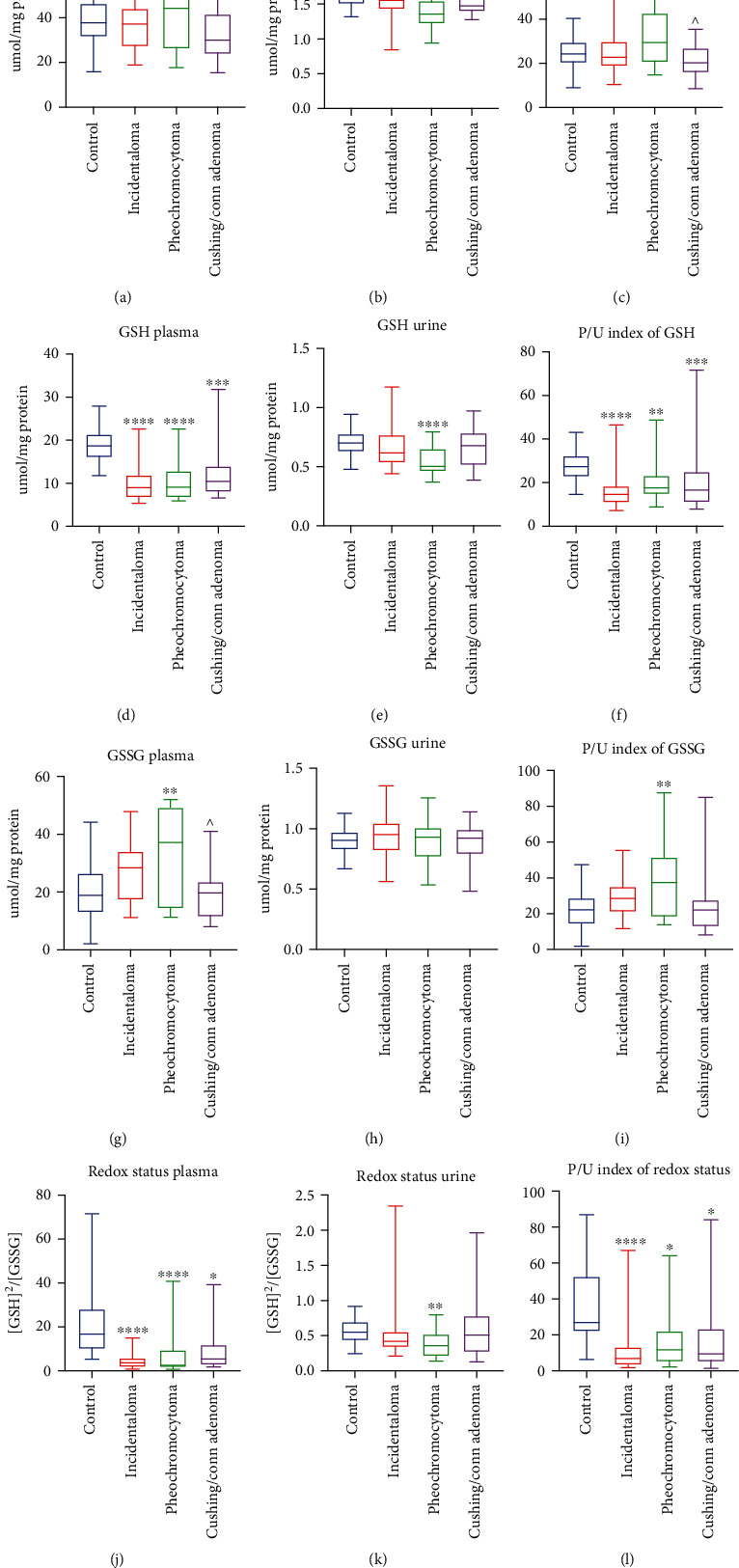
Plasma and urine concentration, as well as plasma/urine index of total glutathione (a–c), reduced glutathione (GSH) (d, e, f), glutathione disulfide (GSSG) (g)–(i), and redox status (j)–(l) of the controls, incidentaloma, pheochromocytoma, and Cushing's/Conn's adenoma patients. Results are presented as median with minimum and maximum. ^∗^*p* < 0.05, ^∗∗^*p* < 0.01, ^∗∗∗^*p* < 0.001, and ^∗∗∗∗^*p* < 0.0001 indicate significant differences from the controls; ^*p* < 0.05 indicates significant differences from the pheochromocytoma group.

Total glutathione concentration in plasma did not differ between studied groups (Figure 2(a)). We found lower total glutathione concentration only in urine of pheochromocytoma (-12%, *p* = 0.0011) patients than the controls (Figure 2(b)). Moreover, plasma/urine index of GR concentration in Cushing's/Conn's adenoma patients was diminished (-31%, *p* = 0.0299) in comparison with the pheochromocytoma group (Figure 2(c)).

### 3.6. Reduced Glutathione (GSH)

The plasma concentration of GSH, as well as plasma/urine index of GSH concentration, were decreased of all studied groups: incidentaloma (-53%, *p* < 0.0001; -44%, *p* < 0.0001), pheochromocytoma (-52%, *p* < 0.0001; -33%, *p* = 0.0059), and Cushing's/Conn's adenoma (-27%, *p* = 0.0001; -3%, *p* = 0.0008) as compared to the healthy controls (Figures 2(d) and 2(f)), while urine concentration of GSH was lower only in pheochromocytoma patients (-29%, *p* < 0.0001) (Figure 2(e)).

### 3.7. Glutathione Disulfide (GSSG)

The plasma concentration of GSSG was significantly higher only in patients with pheochromocytoma (+95%, *p* = 0.0079) compared to the controls. Additionally, in Cushing's/Conn's adenoma group, GSSG plasma concentration was lower (-46%, *p* = 0.0299) than the pheochromocytoma group (Figure 2(g)). We did not find any differences in urine concentration of GSSG (Figure 2(h)). The plasma/urine index of GSSG concentration was greater in pheochromocytoma (+68%, *p* = 0.0071) than the controls (Figure 2(i)).

### 3.8. Redox Status

We observed that plasma redox status was significantly diminished in all studied groups: incidentaloma (-78%, *p* < 0.0001), pheochromocytoma (-85%, *p* < 0.0001), and Cushing's/Conn's adenoma (-68%, *p* = 0.0111) as compared to the healthy controls. Interestingly, the plasma redox status was the most decreased in the pheochromocytoma group (Figure 2(j)). In urine, redox status was decreased only in pheochromocytoma (-35%, *p* = 0.0012) (Figure 2(k)). The plasma/urine index of redox status was lower in all patients with adrenal masses: incidentaloma (-74%, *p* < 0.0001), pheochromocytoma (-56%, *p* = 0.0124), and Cushing's/Conn's adenoma (-65%, *p* = 0.0283) than in the controls (Figure 2(l)).

### 3.9. Uric Acid (UA)

**Figure 3:**
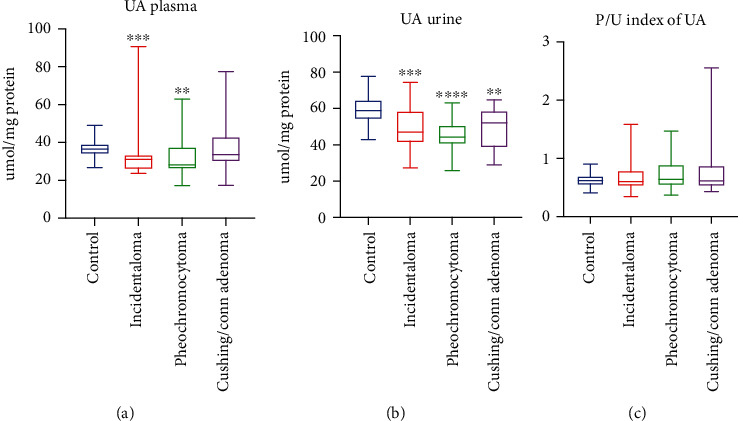
Uric acid (UA) concentration in plasma and urine (a, b), as well as plasma/urine index of UA concentration (c) of the controls, incidentaloma, pheochromocytoma, and Cushing's/Conn's adenoma patients. Results are presented as median with minimum and maximum. ^∗∗^*p* < 0.01, ^∗∗∗^*p* < 0.001, and ^∗∗∗∗^*p* < 0.0001 indicate significant differences from the controls.

The plasma concentration of UA was significantly diminished in incidentaloma (-15%, *p* = 0.0001) and pheochromocytoma (-23%, *p* = 0.0025) groups in comparison with the controls (Figure 3(a)). We observed significantly lower urine concentration of UA in all groups of patients with adrenal masses: incidentaloma (-20%, *p* = 0.0005), pheochromocytoma (-25%, *p* < 0.0001), and Cushing's/Conn's adenoma (-12%, *p* = 0.002) (Figure 3(b)). There were no differences in the plasma/urine index of UA concentration between studied groups (Figure 3(c)).

### 3.10. Advanced Glycation End Products (AGE)

**Figure 4:**
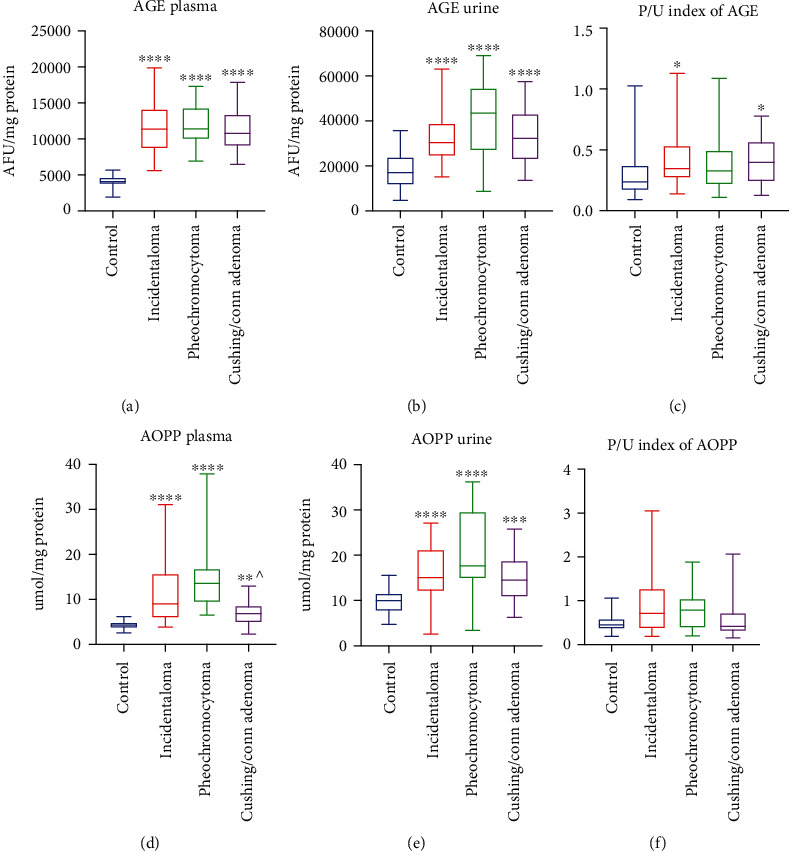
Plasma and urine content of advanced glycation end products (AGE) (a, b) and advanced oxidation protein products (AOPP) (d, e), as well as plasma/urine index of the AGE (c) and AOPP (f) content of the controls, incidentaloma, pheochromocytoma, and Cushing's/Conn's adenoma patients. Results are presented as with minimum and maximum. ^∗^*p* < 0.05, ^∗∗^*p* < 0.01, ^∗∗∗^*p* < 0.001, and ^∗∗∗∗^*p* < 0.0001 indicate significant differences from the controls; ^*p* < 0.05 indicates significant differences from the pheochromocytoma group.

The AGE plasma and urine content were greater in patients with adrenal masses incidentaloma (+179%, *p* < 0.0001; +79%, *p* < 0.0001), pheochromocytoma (+180%, *p* < 0.0001; +157%, *p* < 0.0001), and Cushing's/Conn's adenoma (+165%, *p* < 0.0001; +90%, *p* < 0.0001) in comparison with the controls (Figures 4(a) and 4(b)), while the plasma/urine index of the AGE content was higher in incidentaloma (+46%, *p* = 0.0196) and Cushing's/Conn's adenoma (+67%, *p* = 0.0463) than the controls (Figure 4(c)).

### 3.11. Advanced Oxidation Protein Products (AOPP)

We found a significantly higher plasma and urine concentration of AOPP in all studied groups: incidentaloma (+111%, *p* < 0.0001; +51%, *p* < 0.0001), pheochromocytoma (+217%, *p* < 0.0001; +77%, *p* < 0.0001), and Cushing's/Conn's adenoma (+61%, *p* = 0.0013; +46%, *p* = 0.0006) as compared to the controls. Additionally, patients with Cushing's/Conn's adenoma had lower plasma concentration of AOPP (-49%, *p* = 0.0139) than pheochromocytoma ones (Figures 4(d) and 4(e)). The plasma/urine index of the AOPP content did not differ between studied groups (Figure 4(f)).

### 3.12. Lipid Hydroperoxides (LOOH)

**Figure 5:**
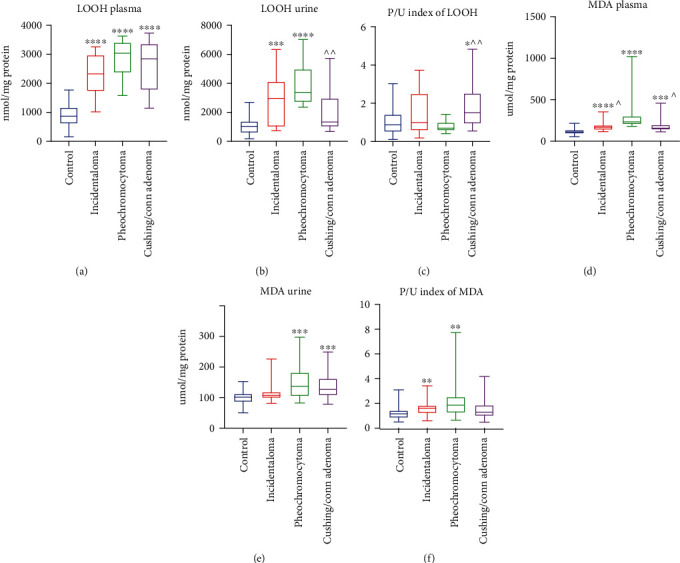
Plasma and urine concentration of lipid hydroperoxides (LOOH) (a, b) and malondialdehyde (MDA) (d, e), as well as plasma/urine index of LOOH (c) and MDA (f) concentration of the controls, incidentaloma, pheochromocytoma, and Cushing's/Conn's adenoma patients. Results are presented as median with minimum and maximum. ^∗^*p* < 0.05, ^∗∗^*p* < 0.01, ^∗∗∗^*p* < 0.001, and ^∗∗∗∗^*p* < 0.0001 indicate significant differences from the controls; ^*p* < 0.05 and ^^*p* < 0.01 indicate significant differences from the pheochromocytoma group.

The patients with adrenal masses had a markedly higher concentration of plasma LOOH than the controls: incidentaloma (+168%, *p* < 0.0001), pheochromocytoma (+249%, *p* < 0.0001), and Cushing's/Conn's adenoma (+227%, *p* < 0.0001) (Figure 5(a)), whereas in urine, the concentration of LOOH was greater in incidentaloma (+184%, *p* = 0.0003) and pheochromocytoma (+223%, *p* < 0.0001) groups. Interestingly, the urine concentration of LOOH in Cushing's/Conn's adenoma patients (-61%, *p* = 0.0075) was lower than in the pheochromocytoma group (Figure 5(b)). Further on, the plasma/urine index of LOOH concentration in Cushing's/Conn's adenoma group was increased in comparison with the controls (+73%, *p* = 0.0135) and pheochromocytoma (+116%, *p* = 0.0076) (Figure 5(c)).

### 3.13. Malondialdehyde (MDA)

The plasma concentration of MDA was increased in patients with adrenal masses as compared to the controls: incidentaloma (+52%, *p* < 0.0001), pheochromocytoma (+114%, *p* < 0.0001), and Cushing's/Conn's adenoma (+42%, *p* = 0.0001). Moreover, we noticed that the MDA plasma concentration in patients with incidentaloma (-29%, *p* = 0.0239) and Cushing's/Conn's adenoma (-33%, *p* = 0.0341) was lower than in the pheochromocytoma group (Figure 5(d)). The urine concentration of MDA was markedly higher in pheochromocytoma (+35%, *p* = 0.0002) and Cushing's/Conn's adenoma (+25%, *p* = 0.0008) groups in comparison with the controls (Figure 5(e)). The plasma/urine index of MDA concentration was greater in incidentaloma (+39%, *p* = 0.0049) and pheochromocytoma (+60%, *p* = 0.0017) than the controls (Figure 5(f)).

### 3.14. DNA/RNA Oxidation Products

**Figure 6:**
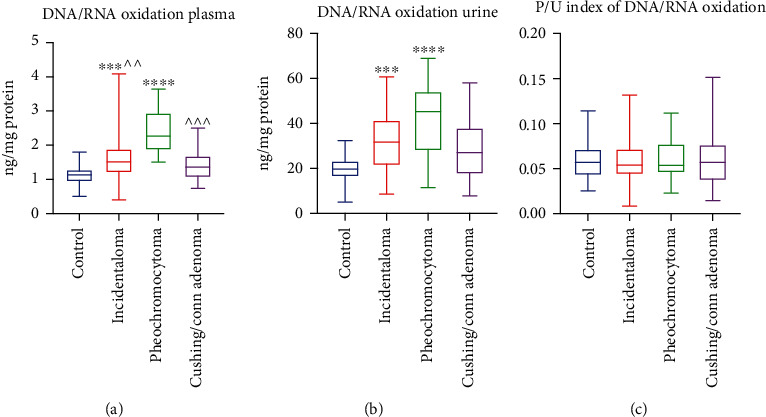
Plasma and urine DNA/RNA oxidation products (a, b) and plasma/urine index of DNA/RNA oxidation product content (c) of the controls, incidentaloma, pheochromocytoma, and Cushing's/Conn's adenoma patients. Results are presented as median with minimum and maximum. ^∗∗∗^*p* < 0.001 and ^∗∗∗∗^*p* < 0.0001 indicate significant differences from the controls; ^^*p* < 0.01 and ^^^*p* < 0.001 indicate significant differences from the pheochromocytoma group.

The DNA/RNA oxidation product content was significantly increased in groups: incidentaloma (+33%, *p* = 0.0005) and pheochromocytoma (+99%, *p* < 0.0001) groups as compared to the controls. Moreover, the DNA/RNA oxidation product content was lower in the incidentaloma (-33%, *p* = 0.0063) and Cushing's/Conn's adenoma groups (-40%, *p* = 0.0004) than the patients with pheochromocytoma (Figure 6(a)). In urine, patients with incidentaloma (+61%, *p* = 0.0001) and pheochromocytoma (+130%, *p* < 0.0001) had greater contents of DNA/RNA oxidation products than the controls (Figure 6(b)). We did not find any significant differences in the plasma/urine index of DNA/RNA oxidation products between any of the groups (Figure 6(c)).

We checked the diagnostic usefulness of the assessed redox biomarkers of adrenal masses. The results of ROC analysis are presented in Table 2. We identified a potential diagnostic utility for pheochromocytoma patients for the plasma MDA (sensitivity 95%; specificity 96.67%) and DNA/RNA oxidation products (sensitivity 95%; specificity 96.67%). Moreover, plasma DNA/RNA oxidation products with high sensitivity (80%) and specificity (80%) differentiate patients with pheochromocytoma from those with Cushing's/Conn's adenoma.

### 3.15. Correlations

**Figure 7:**
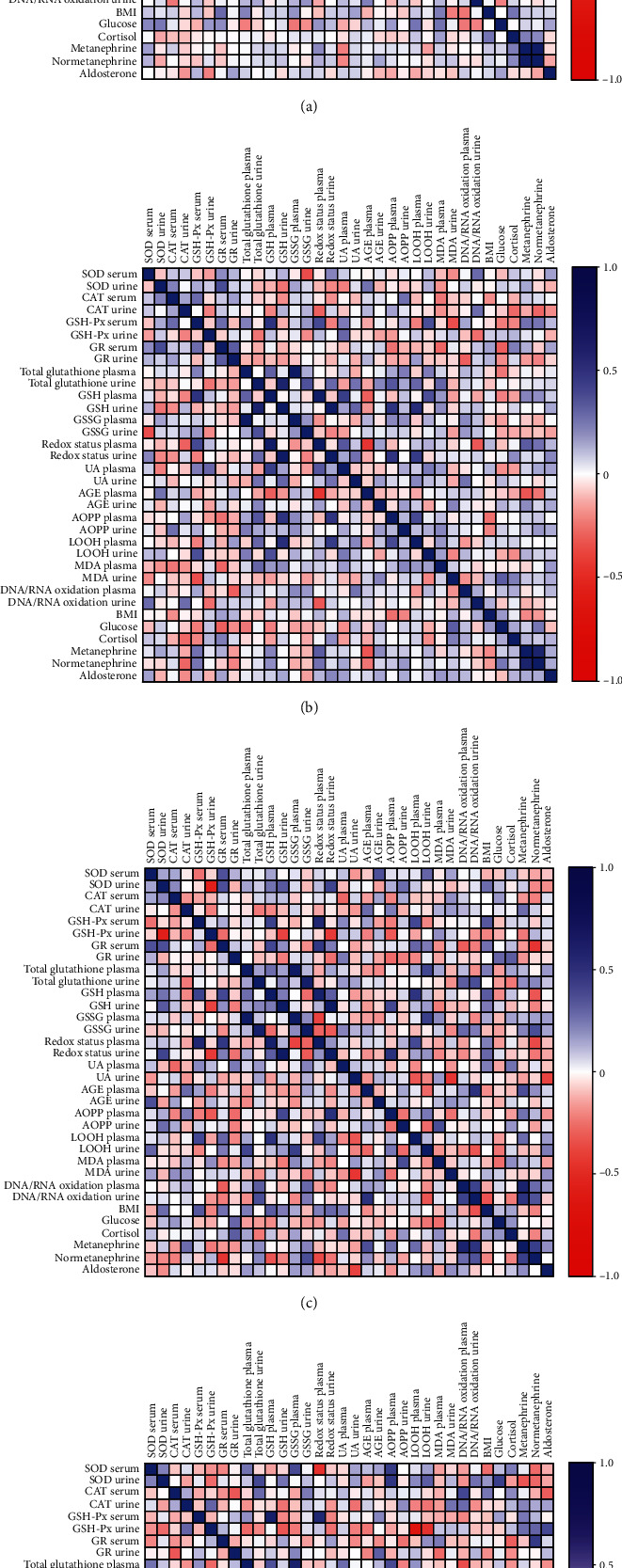
Correlations between the analyzed redox biomarkers and clinical parameters in serum, plasma, and urine of the controls (a) and patients with incidentaloma (b), pheochromocytoma (c), and Cushing's/Conn's adenoma (d). SOD: superoxide dismutase; CAT: catalase; GSH-Px: glutathione peroxidase; GR: glutathione reductase; GSH: glutathione; GSSG: glutathione disulfide; UA: uric acid; AGE: advanced glycation end products; AOPP: advanced oxidation protein products; LOOH: lipid hydroperoxides; MDA: malondialdehyde; BMI: body mass index.

Correlations between the analyzed redox biomarkers and clinical parameters of studied groups are presented in the heat maps (Figure 7).

In the controls, serum aldosterone negatively correlated with urine CAT (*p* = 0.045, *R* = −0.26) and urine GSH-Px (*p* = 0.013, *R* = −0.319). Plasma redox status was associated negatively with serum GSH-PX (*p* = 0.12, *R* = −0.493), plasma total glutathione (p = 0.008, R = -0.521), and plasma GSSG (*p* < 0.0001, *R* = −0.725), whereas positively with plasma GSH (*p* = 0.001, *R* = 0.64). Urine redox status correlated negatively with urine GSSG (*p* < 0.0001, *R* = −4.94) and positively with urine total glutathione (*p* = 0.008, *R* = 0.337) and urine GSH (*p* < 0.0001, *R* = 0.938). We found positive correlations between serum GR and plasma MDA (*p* = 0.006, *R* = 0.349) and BMI (*p* = 0.034, *R* = 0.389), plasma total glutathione and plasma GSH (*p* = 0.015, *R* = 0.314) and plasma GSSG (*p* < 0.0001, *R* = 0.925), urine total glutathione, urine GSH (*p* < 0.0001, *R* = 0.618) and urine GSSG (*p* < 0.0001, *R* = 0.613), and plasma GSSG and plasma UA (*p* = 0.036, *R* = 0.271), as well as serum cortisol, urine metanephrine (*p* = 0.012, *R* = 0.323), and urine normetanephrine (*p* = 0.023, *R* = 0.293). The high positive correlation was also between urine metanephrine and urine normetanephrine (*p* < 0.0001, *R* = 0.881). A negative correlation was also showed between plasma GSH and plasma RNA/DNA oxidation products (*p* = 0.014, *R* = −0.315), plasma UA, urine metanephrine (*p* = 0.012, *R* = −0.322), and urine normetanephrine (*p* = 0.018, *R* = −0.303), as well as urine UA and urine AGE (*p* = 0.013, *R* = −0.318) (Figure 7(a)).

The incidentaloma group serum GSH-Px correlated positively with serum CAT (*p* = 0.032, *R* = 0.392), plasma GSH (*p* = 0.001, *R* = 0.592), plasma redox status (*p* = 0.008, *R* = 0.498), and urine metanephrine (*p* = 0.045, *R* = −0.368). Plasma GSH was associated positively with plasma redox status (*p* < 0.0001, *R* = 0.846), plasma UA (*p* = 0.002, *R* = 0.547), and negatively with plasma AGE (*p* = 0.019, *R* = −0.426). The positive correlations were observed between plasma redox status and plasma UA (*p* = 0.018, *R* = 0.452), urine metanephrine (*p* = 0.021, *R* = 0.442) and urine normetanephrine (*p* < 0.0001, *R* = 0.719), urine MDA and serum glucose (*p* = 0.025, *R* = 0.438), plasma AOPP and plasma LOOH (*p* = 0.006, *R* = 0.492), urine redox status and urine GSH (*p* < 0.0001, *R* = 0.886), and urine total glutathione (*p* = 0.04, *R* = 0.397). Additionally, urine total glutathione correlated positively with urine GSH (*p* < 0.0001, *R* = 0.718), urine GSSG (*p* = 0.009, *R* = 0.471), and urine UA (*p* = 0.047, *R* = 0.366). We also found positive correlations between plasma total glutathione and plasma GSH (*p* = 0.02, *R* = 0.423) and plasma GSSG (*p* < 0.0001, *R* = 0.913), whereas the negative correlations were observed between urine GSH-Px and urine RNA/DNA oxidation products (*p* = 0.044, *R* = −0.37), plasma redox status and plasma AGE (*p* = 0.001, *R* = −0.6), and urine metanephrine and plasma AGE (*p* = 0.012, *R* = −0.454) (Figure 7(b)).

In patients with pheochromocytoma, we observed that plasma redox status correlated positively with serum GSH-Px (*p* = 0.001, *R* = 0.669), serum GR (*p* = 0.008, *R* = 0.576), plasma GSH (*p* < 0.0001, *R* = 0.761), and plasma LOOH (*p* = 0.025, *R* = 0.501), while it correlated negatively with plasma GSSG (*p* = 0.015, *R* = −0.535) and urine normetanephrine (*p* = 0.023, *R* = −0.507). Interestingly, highly positive correlation was found between urine redox status and urine GSH (*p* < 0.0001, *R* = 0.913) and urine SOD (*p* = 0.024, *R* = 0.516), while negative correlation was identified between urine redox status and urine GSH-Px (*p* = 0.011, *R* = −0.572). Additionally, urine GSH-Px was associated negatively with urine SOD (*p* < 0.0001, *R* = −0.725) and urine GSH (*p* = 0.019, *R* = −0.531). Negative correlations were also observed between serum GR and urine normetanephrine (*p* = 0.005, *G* = −0.597), plasma GSH and urine normetanephrine (*p* = 0.045, *R* = −0.453), urine UA and urine MDA (*p* = 0.007, *R* = −0.595), and urine UA and serum aldosterone (*p* = 0.024, *R* = −0.529). We found positive correlation between serum GR and serum SOD (*p* = 0.038, *R* = 0.468) and plasma GSH (*p* = 0.023, *R* = 0.507), as well as plasma total glutathione and plasma GSSG (*p* < 0.0001, *R* = 0.886), whereas urine total glutathione correlated positively with urine GSSG (*p* < 0.0001, *R* = 0.786) and urine RNA/DNA oxidation products (*p* = 0.032, *R* = 0.48). The positive correlation were also identified between plasma GSH and plasma LOOH (*p* = 0.005, *R* = 0.603), urine GSSG and urine normetanephrine (*p* = 0.032, *R* = 0.48), urine RNA/DNA oxidative products and urine normetanephrine (*p* = 0.039, *R* = 0.465), plasma AGE and urine metanephrine (*p* = 0.033, *R* = 0.478). Moreover, urine metanephrine was associated positively with plasma and urine RNA/DNA oxidative products (*p* < 0.0001, *R* = 0.714; *p* = 0.005, *R* = 0.597) and urine normetanephrine (*p* = 0.014, *R* = 0.541) (Figure 7(c)).

In Cushing's/Conn's group, we found positive correlations between serum SOD and plasma GSSG (*p* = 0.026, *R* = 0.498), urine SOD and serum glucose (*p* = 0.02, *R* = 0.542), plasma GSH and plasma GSH-Px (*p* < 0.0001, *R* = 0.785), serum GR and urine normetanephrine (*p* = 0.009, *R* = 0.565), plasma total glutathione and plasma GSH (*p* = 0.038, *R* = 0.468), plasma total glutathione and plasma GSSG (*p* < 0.0001, *R* = 0.749), and urine total glutathione and urine GSSG (*p* = 0.004, *R* = 0.617), as well as urine UA and urine RNA/DNA oxidative products (*p* = 0.001, *R* = 0.662). Urine RNA/DNA oxidative products correlated positively with urine LOOH (*p* = 0.024, *R* = 0.502). Plasma redox status was associated positively with GSH-Px (*p* = 0.002, *R* = 0.674) and plasma GSH (*p* < 0.0001, *R* = 0.798). Similarly, urine plasma redox status correlated highly positive with urine GSH (*p* < 0.0001, *R* = 0.974) and negative with urine GSSG (*p* = 0.001, *R* = −0.695). The negative correlations were observed between urine SOD and urine metanephrine (*p* = 0.042, *R* = −0.459), plasma total glutathione and plasma RNA/DNA oxidative products (*p* = 0.029, *R* = −0.487), and urine metanephrine and BMI (*p* = 0.008, *R* = −0.571) (Figure 7(d)).

## 4. Discussion

One of the most important factors involved in the development of neoplasms is oxidative stress. This process initiates DNA damage and leads to genetic mutations and chromosomal instability [18, 22, 36]. In biological systems, ROS play a dual role, both beneficial and harmful. ROS's positive effects include the cellular response against infectious agents and participation in cell signaling as messengers' factors. Low concentrations of free radicals also induce a mitogenic response [37, 38]. However, enhanced formation of ROS leads to oxidative damage to cellular structures, and therefore, disrupts the cell's metabolism. Indeed, ROS overproduction and altered regulation of redox-related signaling pathways have been observed in various types of cancer [22, 39]. The carcinogenesis process is associated with DNA oxidative damage, which in turn results in replication errors, genome instability, and impaired signal transduction pathways [40, 41]. This may be due to depletion of antioxidant reserves; although in some types of cancer, the antioxidant barrier is strengthened as an adaptive response to ROS overproduction [14–17]. Unfortunately, the role of the antioxidant barrier and oxidative stress is not completely understood in the context of adrenal tumors.

This is the first study evaluating the redox balance, glutathione metabolism, and oxidative damage to RNA/DNA, proteins, and lipids in the plasma/serum and urine of patients with adrenal masses. We demonstrated disturbances in enzymatic and nonenzymatic antioxidant barrier: serum SOD and CAT and plasma UA and GSH significantly decreased with simultaneous increases of blood GSH-Px and GSSG in patients with adrenal masses. Moreover, we observed a greater amount of oxidative damage products of RNA/DNA, proteins (↑AGE, ↑AOPP), and lipids (↑LOOH, ↑MDA) in the plasma and urine in these patients. Most redox biomarkers did not differentiate study groups: incidentaloma, pheochromocytoma, and Cushing's/Conn's adenoma. Nevertheless, we found that plasma RNA/DNA oxidation products could differentiate pheochromocytoma and Cushing's/Conn's adenoma with high specificity and sensitivity.

Cells of aerobic organisms have evolved many defense mechanisms to protect themselves from ROS overproduction. In our study, we generally showed a reduction in the antioxidant defense capacity (serum/plasma: ↓SOD, ↓CAT, ↓GSH, ↓UA; urine: ↓SOD, ↓GSH, ↓UA), which may be responsible for the enhanced oxidation of proteins, lipids, and DNA at a systemic level. This can be confirmed by the negative correlation between plasma total glutathione and plasma RNA/DNA oxidative products, urinary GSH-Px and RNA/DNA oxidation, and plasma redox status and plasma AGEs. Of particular note is the decrease in GSH, the major intracellular nonenzymatic antioxidants, with a concomitant increase in its oxidized form (GSSG). Glutathione disulfide is highly toxic to the body because it enhances protein glutathiolation and induces cell death by apoptosis or necrosis. Thus, GSSG disrupts the thiol status of the cell affecting the regulation of gene transcription, enzyme activity, and expression of various cell receptors [42, 43]. Because the main defense mechanism against GSSG overload is its translocation outside the cells [44, 45], the increase in plasma oxidized glutathione may, in part, reflect glutathione metabolism in the cancer cell. Additionally, increased GSH-Px activity in pheochromocytoma patients with a concomitant decrease in CAT activity may indicate their compensatory action in inactivating hydrogen peroxide. H_2_O_2_ does not have a strong oxidizing effect directly, but it readily crosses cell membranes and, together with the superoxide radical, can be a source of the highly reactive hydroxyl radical [39, 46]. Hydrogen peroxide also plays a key role in the regulation of cell proliferation and cell death. Depending on its concentration, a cell can either divide or undergo apoptosis and necrosis [47]. Based on studies of other cancers, it has been suggested that cells with low SOD and CAT activity profile and with variable GSH-Px activity promote cancer tumor formation [48]. However, these mechanisms are not well understood, and there is a lack of any research in the context of adrenal tumors.

Oxidative stress is involved not only in the initiation and promotion of carcinogenesis but also in the tumor progression. Oxidative stress has been shown to increase inflammation and cytokine activity [13, 17]. It is also responsible for intense cancer cell metabolism associated with continuous tumor proliferation, mitochondrial DNA mutations, and mitochondrial dysfunction. Because the largest amounts of ROS are generated in the respiratory chain, the mitochondrial membrane is the most vulnerable to oxidation. As a result of this damage, cytochrome c is released, and the apoptotic cascade is activated [49, 50]. Although most adrenal tumors are benign, it is still unclear whether they can become malignant and what factors may influence tumor metastasis. Although our study does not explain this, disturbances in the antioxidant barrier might promote or enhance the process of adrenal tissue transformation into a tumor [51]. Mechanisms responsible for enhancing cellular proliferation may include direct interaction of free radicals with specific receptors and modulation of the expression of important signaling agents, such as protein kinases and transcription factors [51, 52]. In pheochromocytomas, the major genetic aberrations involve kinase signaling and protein translation genes [53, 54]. It is well known that under oxidative stress conditions, there are upregulation of src/Abl kinase, PI3 kinase, and MAPK dependent signaling pathways as well as activation of redox-regulated transcription factors such as NF-*κ*B, AP-1, p53, NFAT, and HIF-1 [22]. In pheochromocytoma, the most common mutations occur in genes involved in the VHL/HIF axis, including PHD, VHL, and HIF [53]. HIFs are transcription factors that serve as major regulators of oxygen metabolism [55, 56]. They have various effects on tumor growth affecting cell proliferation, differentiation, vascularization, angiogenesis, tumor immune response, invasion, metastasis, and apoptosis [57]. HIFs are also the main transcription factors responding to hypoxia in the cell [58]. Interestingly, the increased HIF-1*α* expression contributes to mitochondrial dysfunction and ROS overproduction [59–61]. Indeed, HIF-1 activation by stabilization of HIF-1*α* upregulates NADPH oxidase, which is the main source of ROS in response to hypoxia [62, 63]. Reduced GSH levels increase the synthesis of inflammatory mediators (such as IL-1*β* and TNF-*α*), which in turn induce the synthesis of HIF-1*α* [64]. Although the involvement of HIF and oxidative stress in adrenal tumors seems to be important, our hypotheses need to be verified in further molecular studies.

Pheochromocytoma can be classified as a metabolic disease due to the increased secretion of catecholamines such as dopamine, adrenaline, and noradrenaline. Indeed, catecholamines are involved in the regulation of many metabolic pathways, and therefore, patients with phaeochromocytoma may have impaired glucose metabolism, insulin resistance, and lipid metabolism disorders [65, 66]. Pheochromocytoma is also a secondary cause of diabetes mellitus, which, in some patients, may be the only clinical symptom of the tumor. Although adrenal tumors are responsible for various metabolic complications, serum glucose levels in our patients are generally within the reference range. The surgical removal of the cancer results in the remission of diabetes, emphasizing the pathogenic role of excess catecholamines [67, 68]. Nevertheless, the development of insulin resistance in patients with adrenal tumors is not clear. This can be related to the decreased levels of adiponectin [69]. Another explanation may be a constant oxidative stress as suggested by our experiment. As many studies have confirmed the key role of redox imbalance in obesity, insulin resistance, and diabetes pathophysiology [9, 10, 70], metabolic disorders in phaeochromocytoma may result from disturbances in antioxidant barrier and intensification of oxidative stress. This may be indicated by the positive correlation between plasma glucose and urinary MDA levels, as well as between metanephrine and BMI. Although catecholamines may show antioxidant and antiglycation properties, the accumulation of their oxidation products in tissues may have cytotoxic and mutagenic effects [71, 72]. It has been shown that semiquinone radicals (formed in the oxidation of dopamine, epinephrine, and norepinephrine) cause glutathione oxidation as well as induce lipoperoxidation and oxidative DNA damage [73]. Catabolism of catecholamines (mediated by MAO and COMT enzymes) may also exacerbate oxidative stress level in the cell [73]. In general, the concentrations of protein, lipid, and DNA oxidation products were the highest in patients with pheochromocytoma (compared with other tumors), indicating that redox homeostasis is most disturbed in these group of patients. We cannot exclude that obesity and metabolic disorders are the source of impaired redox balance in our patients; however, the highest severity of oxidative stress was observed in patients with pheochromocytoma, in whom body weight is slightly elevated and glucose level is within the reference range.

The diagnosis of adrenal tumors requires complex studies; so in our study, we also decided to assess the markers of oxidative stress in the serum/plasma and urine as a material for research in an easily accessible and minimally invasive manner. It has recently been emphasized that oxidative stress plays a critical role in the pathogenesis of various diseases such as neurodegenerative diseases [74, 75], insulin resistance [76], diabetes [77, 78], hypertension [79, 80], metabolic syndrome [10, 81], stroke [82, 83], and cancer [84, 85]. Thus, we compared whether antioxidants/oxidation products can differentiate between patients with adrenal masses (incidentaloma, pheochromocytoma, and Cushing/Conn adenoma) as well as healthy controls. We have demonstrated that the assessment of plasma MDA and DNA/RNA oxidation products, with high sensitivity and specificity, can help to diagnose pheochromocytoma. Interestingly, plasma DNA/RNA oxidation products can differentiate patients with pheochromocytoma from Cushing/Conn adenoma patients as well as from healthy controls. The obtained correlations confirm the diagnostic usefulness of DNA/RNA oxidation products in patients with pheochromocytoma. Indeed, plasma RNA/DNA oxidative products were positively associated with urine metanephrine, whereas urine RNA/DNA oxidative products positively correlated with metanephrine and normetanephrine. Despite the observed changes in the urine redox biomarkers, we did not find them useful in diagnosing adrenal masses. This may be because the activity/concentration of antioxidants and oxidation products is significantly higher in plasma/serum than in urine, with the exception of DNA/RNA oxidation products, which are excreted via the renal route [86, 87].

The limitation of our study is that we have assessed redox homeostasis and oxidative stress only at the system level. Thus, in further research, it is necessary to evaluate molecular redox mechanisms in adrenal tumor development. Although oxidative stress in patients with adrenal tumors may be due to associated metabolic disorders, it is important to note that the primary cause of these abnormalities is the tumor. Nevertheless, this study is the first to assess redox balance, glutathione metabolism, and oxidative damage to RNA/DNA, proteins, and lipids in the plasma/serum and urine of patients with adrenal masses. It is also worth emphasizing that we have conducted studies on a relatively large number of patients, selectively divided on the type of adrenal tumor.

## 5. Conclusions

1. Patients with adrenal tumors have impaired enzymatic and nonenzymatic antioxidant systems as well as increased oxidative damage to proteins, lipids, and DNA/RNA in both plasma, serum, and urine compared to controls. Antioxidant supplementation may be considered in patients with adrenal masses
2. Plasma DNA/RNA oxidation products can differentiate patients with pheochromocytoma from Cushing's/Conn's adenoma as well as from healthy controls
3. Oxidative stress may play a crucial role in adrenal tumors. Nevertheless, further studies are required to clarify the role of redox signaling in tumor development

## Figures and Tables

**Table 1:** Clinical and laboratory characteristics of the controls, incidentaloma, pheochromocytoma, and Cushing's/Conn's adenoma patients. Results are presented as median with 25% and 75% percentiles. ^∗^*p* < 0.05, ^∗∗^*p* < 0.01, ^∗∗∗^*p* < 0.001, and ^∗∗∗∗^*p* < 0.0001 indicate significant differences from the controls; ^*p* < 0.05, ^^^^*p* < 0.0001 indicates significant differences from the pheochromocytoma group; ~*p* < 0.05 indicates significant differences from Cushing's/Conn's adenoma group. BMI: body mass index; HGB: hemoglobin; PLT: platelet count; RBC: red blood cell count; WBC: white blood cell count.

	Controls (*n* = 60)	Incidentaloma (*n* = 30)	Pheochromocytoma (*n* = 20)	Cushig's/Conn's adenoma (*n* = 20)	ANOVA
Age	59 (50-65)	61 (54-65)	56 (50-65)	59 (53-65)	*p* = 0.6506
BMI (kg/m^2^)	23.04 (22.6-23.82)	29.62^∗∗∗∗^ (26.13-34.98)	26.75^∗^ (22.58-31.97)	30.57^∗∗∗∗^ (27.1-32.5)	*p* < 0.0001
Glucose (mg/dl)	76 (72-82.25)	101^∗∗∗^ (89.5-113.5)	84.5^∗^ (79.25-104.3)	97.5^∗∗∗^ (82.5-110)	*p* < 0.0001
Na^+^ (mmol/l)	139 (137-140)	140 (138-141)	139.5 (137-141)	138 (136-141)	*p* = 0.1764
K^+^ (mmol/l)	4.39 (4.15-4.66)	4.51 (4.29-4.81)	4.28 (4.06-4.69)	4.32 (3.78-4.65)	*p* = 0.1333
WBC (10^3^/*μ*l)	7.5 (6.8-8.3)	7.04 (5.265-8.8)	7.51 (6.428-8.743)	7.27 (6.038-9.17)	*p* = 0.7462
RBC (10^6^/*μ*l)	4.6 (4.5-4.9)	4.74 (4.35-4.98)	4.51 (4.135-4.808)	4.625 (4.385-4.81)	*p* = 0.3823
HGB (g/dl)	13.8 (13.4-14.1)	14.2 (13.5-14.7)	14.1 (12.7-14.9)	14.1 (13.3-14.5)	*p* = 0.2541
PLT (10^3^/*μ*l)	289 (275-300)	225^∗∗∗∗^ (183.5-270.5)	264.5 (215-285.3)	195^∗∗∗∗^^ (165-237)	*p* < 0.0001
Serum cortisol before 10 a.m. (*μ*g/dl)	11 (9.2-16)	15 (11-19)	15 (11-20)	12 (10-20)	*p* = 0.0506
Urine methanephrine (*μ*g/24 h)	118 (87-208)	98^^^^ (78-176)	592^∗∗∗∗^ (425-862)	122.5^^^^ (94-223)	*p* < 0.0001
Urine normethanephrine (*μ*g/24 h)	234 (173-308)	233^^^^^~^ (174-331)	655^∗∗∗^ (553-934)	394^∗∗^^ (271-435)	*p* < 0.0001
Aldosterone (ng/dl)	12.3 (8.63-20.18)	12.08^~^ (6.8-19.56)	16.3 (10.82-21.88)	18.56^∗^ (11.56-35.8)	*p* = 0.0195
Size of the tumor (cm)		4.2 (2.65-5.5)	3.8 (3.5-4.8)	3.45 (2.025-4.95)	*p* = 0.4155

**Table 2:** Area under the curve (AUC) of malondialdehyde (MDA) and DNA/RNA oxidation products between the controls and incidentaloma and pheochromocytoma, as well as Cushing's/Conn's adenoma patients, pheochromocytoma and incidentaloma patient, pheochromocytoma and Cushing's/Conn's adenoma patients, incidentaloma and Cushing's/Conn's adenoma patients.

	AUC	95% CI	*p* value	Cutoff	Sensitivity %	95% CI	Specificity %	95% CI
Controls vs. incidentaloma								
MDA	0.8594	0.7856 to 0.9333	<0.0001	> 135.2	76.67	59.07% to 88.21%	75	62.77% to 84.22%
DNA/RNA oxidation products	0.7694	0.6535 to 0.8853	<0.0001	> 1.255	73.33	55.55% to 85.82%	71.67	59.23% to 81.49%
Controls vs. pheochromocytoma								
MDA	0.99	0.9732 to 1.000	<0.0001	> 180.2	95	76.39% to 99.74%	96.67	88.64% to 99.41%
DNA/RNA oxidation products	0.9908	0.9765 to 1.000	<0.0001	> 1.587	95	76.39% to 99.74%	95	86.30% to 98.64%
Controls vs. Cushing's/Conn's adenoma								
MDA	0.8483	0.7636 to 0.933	<0.0001	> 136.7	75	53.13% to 88.81%	76.67	64.56% to 85.56%
DNA/RNA oxidation products	0.6842	0.5380 to 0.8303	0.0141	> 1.185	60	38.66% to 78.12%	58.33	45.73% to 69.94%
Pheochromocytoma vs. incidentaloma								
MDA	0.8467	0.7400 to 0.9533	<0.0001	> 201.2	75	53.13% to 88.81%	76.67	59.07% to 88.21%
DNA/RNA oxidation products	0.825	0.7079 to 0.9421	0.0001	> 1.927	75	53.13% to 88.81%	76.67	59.07% to 88.21%
Pheochromocytoma vs. Cushing's/Conn's adenoma								
MDA	0.8325	0.6923 to 0.9727	0.0003	< 201.2	75	53.13% to 88.81%	75	53.13% to 88.81%
DNA/RNA oxidation products	0.9025	0.8086 to 0.9964	<0.0001	< 1.752	80	58.40% to 91.93%	80	58.40% to 91.93%
Incidentaloma vs. Cushig's/Conn's adenoma								
MDA	0.525	0.3575 to 0.6925	0.7664	< 161.7	55	34.21% to 74.18%	53.33	36.14% to 69.77%
DNA/RNA oxidation products	0.5983	0.4371 to 0.7596	0.2427	< 1.471	55	34.21% to 74.18%	56.67	39.20% to 72.62%

## Data Availability

The article contains complete data used to support the findings of this study.
